# Computed Tomography of Aortic Wall Calcifications in Aortic Dissection Patients

**DOI:** 10.1371/journal.pone.0102036

**Published:** 2014-07-08

**Authors:** Pim A. de Jong, Willem E. Hellings, Richard A. P. Takx, Ivana Išgum, Joost A. van Herwaarden, Willem P. Th. M. Mali

**Affiliations:** 1 Department of Radiology, University Medical Center Utrecht, Utrecht, The Netherlands; 2 Department of Vascular Surgery, University Medical Center Utrecht, Utrecht, The Netherlands; 3 Images Sciences Institute, University Medical Center Utrecht, Utrecht, The Netherlands; University Medical Center (UMC) Utrecht, Netherlands

## Abstract

**Objectives:**

To investigate the frequency of aortic calcifications at the outer edge of the false lumen and the frequency of fully circular aortic calcifications in a consecutive series of patients with aortic dissection who underwent contrast-enhanced CT.

**Methods:**

The study population compromised of 69 consecutive subjects aged 60 years and older with a contrast-enhanced CT scan demonstrating an aortic dissection. All CT scans were evaluated for the frequency of aortic calcifications at the outer edge of the false lumen and the frequency of fully circular aortic calcifications by two experienced observers. Between observer reliability was evaluated by using Cohen’s Kappa. Differences between groups were tested using unpaired T test and Chi-square test.

**Results:**

Presumed media calcifications were observed in 22 (32%) patients of 60 years and older and were found more frequently in chronic aortic dissection (N = 12/23, 52%) than in acute aortic dissection (N = 10/46, 22%).

**Conclusion:**

As the intima has been torn away by the aortic dissection it is highly likely that CT scans can visualize the calcifications in the tunica media of the aorta.

## Introduction

**Figure 1:**
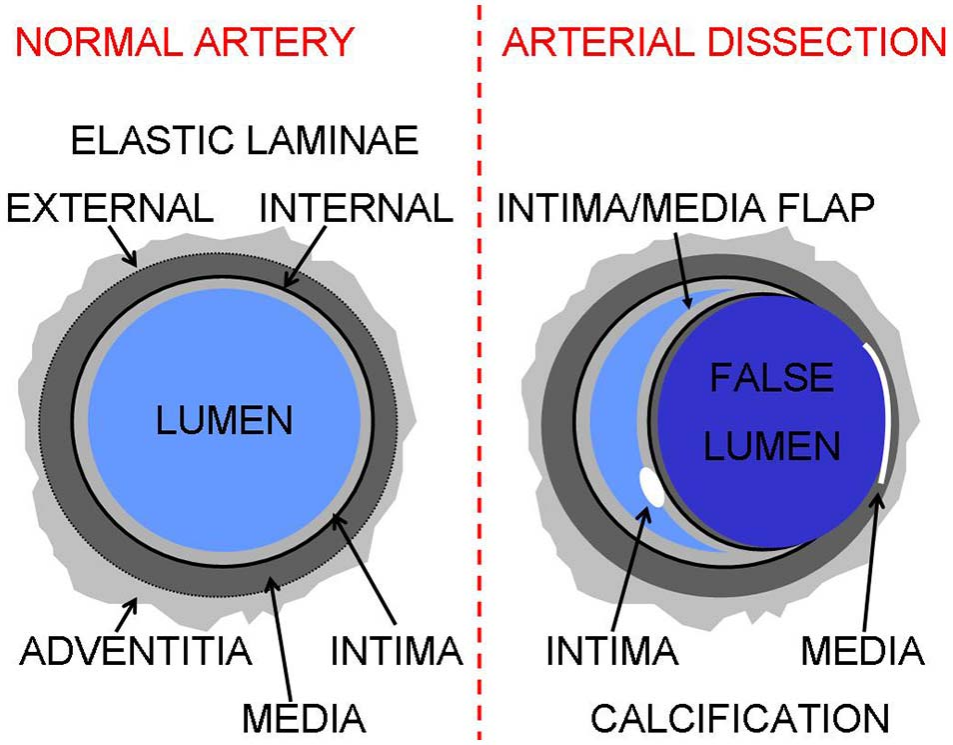
Drawing of a normal and a dissected aorta and location of calcifications.

Aortic dissection is sometimes accompanied with outer wall calcifications in the false lumen [1]. At the edge of the false lumen the intima has been torn away [2] and therefore it is highly likely that these calcifications are either located in the tunica media or in the tunica adventitia (Figure 1). Aortic wall calcifications are increasingly recognized as strong predictors of cardiovascular events and all-cause mortality [3], [4], [5]. It is often thought that all of the calcifications as seen on computed tomography (CT) scans are located in plaques within the (neo-)intima (atherosclerosis) [6]. The active formation of these calcifications in atherosclerotic plaques starts from early adulthood to old age [7], [8], [9]. Pathologists, however, have noted for long that calcifications can be seen in the human aorta in the tunica media before calcifications are seen in neo-intima plaques [10], [11], [12]. Furthermore, pathologic studies suggest that on average at all ages the amount of calcium is higher in the tunica media compared to the intima [13], [14]. Additionally, Juraszek et al. [15] observed a clear association between medial degeneration and type A aortic dissections in patients below 65 years of age, while in those above 65 years type A aortic dissections were associated with atherosclerosis. Thin, mainly circular, wall calcifications in the tunica media are associated with aortic stiffening, increased pulse pressure, reduced coronary blood flow and left ventricular hypertrophy [16], [17], [18]. In the extremities at least part of the media calcifications are hypothesised to be of ‘neurogenic-origin’ closely related to diabetic neuropathy and post-lumbar sympathectomy [19], [20]. Although CT does not have the contrast and resolution to differentiate the tunica media from the intima, Yamada et al. [21] histologically confirmed in the internal carotid artery that calcifications outside of the lumen/plaque were located in the tunica media. Conventional radiographic studies, that have much higher resolution compared to CT, have been accurate in differentiating intima and media calcifications in the extremities [12], [16], [22]. Since the aorta and coronary arteries are located deep in the body, these arteries could not be studied in great detail in vivo with radiography. The potential of distinction between intima and media calcifications in the aorta by CT imaging would provide an opportunity to study the etiology and prognostic value of both types of calcifications.

The aim of this study was to systematically determine the frequency of aortic calcifications at the outer edge of the false lumen and the frequency of fully circular aortic calcifications in a consecutive series of patients with aortic dissection who underwent CT. Since the false lumen of the dissections contains no intima, calcifications observed in the false lumen must be located in the media, especially in acute dissection.

## Materials and Methods

### Subjects

We searched our radiology database for CT scans of the aorta between January 2005 and June 2011. During this period 2235 aortic CT scans were acquired. In the reports of those scans we searched for the key-word dissection, which was mentioned in 599 reports. In 108 patients a dissection was present on the CT scan and in the remainder it was excluded. As we were interested in wall calcifications and as these calcifications occur more frequently with increasing age, subjects below 60 years of age were excluded [23]. The study population therefore compromised 69 consecutive subjects aged 60 years and older with a CT scan demonstrating an aortic dissection. The study protocol was evaluated by the ethical review board of the University Medical Center Utrecht and formal approval and informed consent was waived.

### Clinical data

Patients’ charts were evaluated by one physician. The presence of diabetes, hypertension, dyslipidemia, smoking history or renal dysfunction at the time of the dissection was recorded. Due to the retrospective nature of this study not all measures were available or collected in a structured way for all subjects. Diabetes was defined as physician diagnosed diabetes according to the patient file or use of anti-diabetic drugs. Hypertension was defined as physician diagnosed hypertension according to the patient file or use of anti-hypertensive drugs. Dyslipidemia was defined as physician diagnosed dyslipidemia according to the patient file or use of statins. Smoking was defined as current or former smoker according to the patient file. Renal dysfunction was defined as a glomerular filtration rate below 60 mL/min/1.73 m^2^, being on dialysis or having a renal transplant. Symptomatic aortic dissection or dissection caused by iatrogenic causes was classified as acute dissection. Dissection in patients referred to our tertiary center with a known Stanford B dissection or dissections that were coincidental diagnosed were classified as chronic dissections. Additionally the type of dissection according to the Stanford classification (either Stanford A or B) was recorded.

### CT scanning protocol

As this is a retrospective study and as newer CT technology became available between 2005 and 2011 a variety of CT protocols was used. These protocols can be summarized as follows. Consistently thin-section CT scans were acquired with 16–256 multi-detector-row CT scanners (Philips Medical System, Cleveland, Ohio, USA). In all CT scans intravenous contrast was used to enhance the lumen of the aorta. The CT scans covered chest, abdomen and pelvis and mostly retrospective ECG-gating was applied.

### CT scan evaluation

All CT scans were evaluated by two blinded and independent observers, a senior resident with a special interest in vascular imaging and a chest radiologist with over 10 years experience in chest imaging. In the aorta calcifications were scored as follows. First calcifications at the outer edge of the false lumen were evaluated. If these were absent circular calcification with a longitudinal dimension (length) of more than 1 centimeter were identified. If those were also absent calcifications were judged as non-specific or absent. Furthermore, calcifications were scored as either absent or present in the coronary arteries, splenic artery, aortic valve and mitral valve or mitral annulus. After the blinded and independent reading a consensus reading was done to solve discrepancies in the aortic wall scores.

### Data analysis

Between observer reliability was evaluated by using Cohen’s Kappa. Kappa values were judged as follows: 0.21–0.40 fair reliability, 0.41–0.60 moderate reliability and 0.61–0.80 good reliability. Differences between groups were tested with unpaired T test and Chi-square test. P<0.05 was considered significant. Data are presented as mean and standard deviation or number and percentage.

## Results

### Study population

The majority of subjects were male, the average age was above 70 years, and over half of all subjects had hypertension (Table 1). Diabetes was uncommon and renal dysfunction was present in a quarter. The majority had coronary artery calcifications and nearly half had splenic calcifications (Table 1).

**Table 1:** Characteristics of the study population.

Characteristic	N = 69
Age; mean (SD)	72.6 (7.0)
Gender male; N (%)	50 (72%)
Stanford A; N (%)	37 (54%)
Acute dissection; N (%)	46 (67%)
Diabetes; N (%)	5 (9%); 16 unknown
Hypertension; N (%)	36 (65%); 14 unknown
Smoking; N (%)	11 (22%); 18 unknown
Dyslipidemia; N (%)	9 (18%); 19 unknown
Renal dysfunction; N (%)	15 (25%); 10 unknown
Coronary arteries; N (%)	58 (84%)
Splenic artery; N (%)	30 (43%)
Aortic valve; N (%)	22 (35%); 6 prosthesis
Mitral valve or annulus; N (%)	8 (12%)

### Aortic calcifications

**Figure 2:**
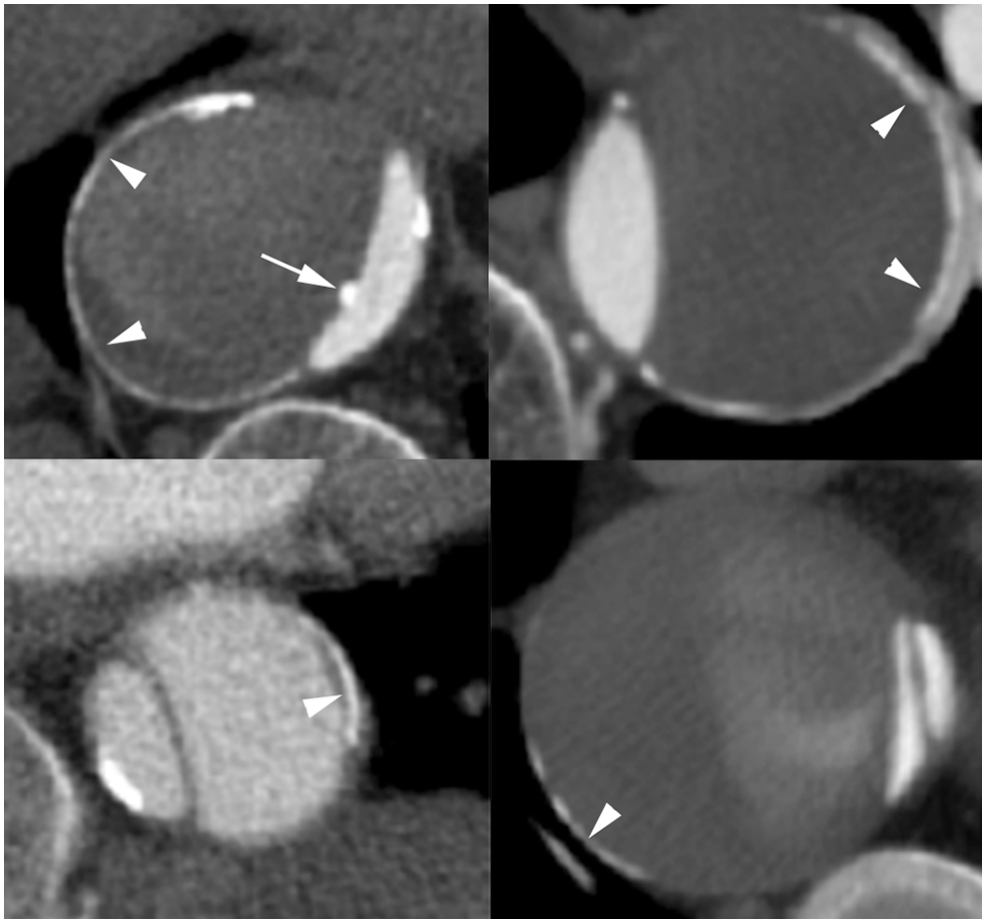
Illustration of patients with aortic dissection who have calcifications at the outer edge of the false lumen. Illustration of four different patients (aged 65–77) with an aortic dissection and thin linear calcifications at the outer edge of the false lumen (arrowheads) where the intima has been torn away. In contrast to the dot like calcifications observed in the intima, these thin mainly circular calcifications are most likely located in the media or adventitia layer of the aortic wall. Note the courser calcification at the intima flap (arrow) presumed to be an intima calcification related to atherosclerosis.

Calcifications at the outer edge of the false lumen were seen in 18 patients and another 4 patients had circular aortic calcifications over a length of >1 cm. Those were grouped as presumed media calcifications (N = 22/69, 32%). Presumed media calcifications were found more frequently in chronic aortic dissection (N = 12/23, 52%) than in acute aortic dissection (N = 10/46, 22%). The calcifications at the outer edge of the false lumen are illustrated in Figure 2. In comparison with the remainder of the patients who did not fulfil the strict criteria; the group with presumed media calcifications was on average 3.7 years older (p = 0.04) and less often male (p = 0.04; Table 2). The presumed media calcifications were not significantly associated with diabetes or renal dysfunction. In the group with presumed media calcifications splenic calcifications (p = 0.005) and calcifications on the mitral valve or annulus (p<0.0001) were recorded more often (Table 3). Kappa values between the two observers were fair to good. Kappa values were for the type of aortic wall calcifications 0.31, presence of coronary artery calcifications 0.34, presence of splenic artery calcifications 0.72, presence of aortic valve calcifications 0.56, and presence of mitral valve or annulus calcifications 0.64.

**Table 2:** Differences in clinical characteristics between aortic dissection patients with and without media calcifications.

	Presumed media calcifications in the aorta (N = 22)	Non-specific or no calcifications in the aorta (N = 47)	P-value for difference
Age; mean (SD)	75.1 (5.9)	71.4 (7.2)	0.04
Gender male; N (%)	12 (55%)	38 (81%)	0.04
Stanford A; N (%)	10 (45%)	27 (57%)	0.44
Acute dissection; N (%)	10 (45%)	36 (77%)	0.02
Diabetes; N (%)	0 (0%); 4 unknown	5 (14%); 12 unknown	0.18
Hypertension; N (%)	13 (72%); 4 unknown	23 (62%); 10 unknown	0.73
Smoking; N (%)	4 (22%); 4 unknown	7 (21%); 14 unknown	0.59
Dyslipidemia; N (%)	3 (19%); 6 unknown	6 (18%); 13 unknown	0.99
Renal dysfunction; N (%)	4 (21%); 3 unknown	11 (28%); 7 unknown	0.86

Differences are tested with unpaired samples T-test (age) or Chi-square (other variables).

**Table 3:** Calcifications of other cardiovascular structures in aortic dissection patients with and without media calcifications.

Calcification in:	Presumed media calcifications in the aorta (N = 22)	Non-specific or no calcifications in the aorta (N = 47)	P-value for difference
Coronary arteries; N (%)	21 (95%)	37 (79%)	0.07
Splenic artery; N (%)	15 (68%)	15 (32%)	0.005
Aortic valve; N (%)	7 (38%); 3 prosthesis	15 (34%); 3 prosthesis	0.53
Mitral valve or annulus; N (%)	7 (32%)	1 (2%)	<0.001

Differences are tested using Chi-square tests.

## Discussion

We show that calcifications at the outer edge of the false lumen of an aortic dissection and/or fully circular aortic calcifications are seen on CT scans in one-third of aortic dissection patients of 60 years and older. Since the intima has been torn away at this part of the aortic wall it is highly likely that CT scans can visualize the thin linear calcifications in the tunica media of the aorta that have been described by pathologists many years ago. Moreover, we also demonstrate that these presumed media calcifications are not only present in chronic aortic dissection [1] but also in acute aortic dissection.

Being intrigued by a patient with a circular thin line of calcium in the aortic wall at the outer side of the false lumen of an aortic dissection, we decided to systematically evaluate a series of dissection patients and demonstrate that this finding is not a rare occurrence. Our findings are supported by previous work from pathologists both in humans and different animals including elephants, cows, dogs and kangaroos [12], [24], [25], [26]. In 1944 Blumenthal et al. [27] studied the relation between age and the amount of calcium in the intima and media of 540 human aortic specimens. The rationale for their study at that time was an apparent overemphasis on intima changes and cholesterol in the pathogenesis of cardiovascular disease. They found that calcifications in the media were more common than in the intima at all ages and that media calcification started in some subjects already before the age of 20 years. At some age-groups hypertensive subjects had more media calcifications and patients with syphilitic aortitis had substantially less media calcifications. Several decades ago these detailed observations have been confirmed by Elliott et al. [28] in 58 aorta’s, by Reid et al. [29] in 128 aorta’s and by Tohno et al. [14] in 23 aorta’s. There is now good evidence that media calcifications are actively formed in a process that resembles membranous bone formation [30], [31], [32].

Our data suggest that, at least in subjects older than 60 years of age, CT scans can visualize aortic calcifications that are highly likely to be in the tunica media. Although we have no pathological proof, we applied strict imaging criteria in combination with a model of aortic dissection that aids separate analysis of the intima and media. In the extremities radiographic criteria of media and intima calcifications have been established in radiology pathology correlation studies. Orr et al. [12] found the following criteria to be highly accurate in differentiating intima and media calcifications on radiography. They defined intima calcifications are irregular, discontinuous, patchy and/or dense clumps and media calcifications as regular, diffuse, fine-grained and/or circumferential. We used the circumferential criterion, which is probably the most rigorous definition.

Recognition of media calcifications in the aortic wall on CT scans can have important clinical and scientific implications. The etiology and prognostic implications of intima and media calcifications are likely to be different. Unraveling the etiology of media calcifications can provide novel therapeutic targets. However, our findings need pathological validation. Also further studies need to concentrate on discriminatory capacity of CT between intima and media calcifications at younger age. In our small series we did not observe an obvious relationship of the presumed media calcifications with diabetes or renal dysfunction that are thought to be the main drivers, next to aging.

Our study is limited by its retrospective nature, small number of patients, and by the fact that we had to rely on hospital files for clinical information. Nevertheless it was not our aim to unravel the pathophysiology of these calcifications. Based on our study one cannot conclude that diabetes and renal dysfunction are not important in the development of calcifications in the tunica media of the aorta. Another limitation is the lack of pathological proof. Although in our opinion aortic dissection was a good model to start with, further radiology-pathology correlation studies are essential. Finally, as we used strict criteria for possible media calcifications, the prevalence in aortic dissection patients is likely underestimated.

In conclusion, we used aortic dissection patients as a model to investigate the ability of CT to visualise calcifications in the tunica media of the aorta in vivo. We conclude that it is highly likely that CT can visualize calcifications in the media of the aorta and may therefore, after further pathological validation, become an important method to study the prevalence, etiology and prognostic significance of these aortic media calcifications.
